# Anxiety, Stress and Depression in COVID-19 Survivors From an Italian Cohort of Hospitalized Patients: Results From a 1-Year Follow-Up

**DOI:** 10.3389/fpsyt.2022.862651

**Published:** 2022-06-17

**Authors:** Carla Gramaglia, Eleonora Gattoni, Eleonora Gambaro, Mattia Bellan, Piero Emilio Balbo, Alessio Baricich, Pier Paolo Sainaghi, Mario Pirisi, Valeria Binda, Alessandro Feggi, Amalia Jona, Debora Marangon, Pierluigi Prosperini, Patrizia Zeppegno

**Affiliations:** ^1^Università del Piemonte Orientale UPO, Novara, Italy; ^2^Azienda Ospedaliero Universitaria Maggiore della Carità, Novara, Italy

**Keywords:** COVID-19, psychiatrics sequelae, anxiety, depression, distress

## Abstract

**Background:**

Mental health-related symptoms can persist over time beyond the most common respiratory clinical features of COVID-19. A recent meta-analysis underlined that mental health sequalae may be relevant for COVID-19 survivors and reported the following prevalence rates: 20% for post-traumatic stress disorder, 22% for anxiety, 36% for psychological distress, and 21% for depression. In the context of a multi-disciplinary follow-up project, we already investigated the mid-term (4 months) psychiatric outcomes in a sample of COVID-19 survivors. Patients were re-assessed after 1-year since hospital discharge.

**Methods:**

Follow-up conducted after 1 year involved 196 individuals recovered from COVID-19. Patients were assessed with a multi-disciplinary approach; including both a clinical interview performed by an experienced psychiatrist, trained in the use of the Mini-International Neuropsychiatric Interview (MINI) to assess the presence of anxiety, stress, and depressive symptoms and the following self-administered questionnaires: Beck Anxiety Inventory, Beck Depression Inventory-II, Resilience Scale for Adults, Impact of Event Scale, and COVID-19 Peritraumatic Distress Index (CPDI).

**Results:**

Anxiety (*p* < 0.0001) and depressive (*p* < 0.0003) symptoms registered at the clinical interview showed a significant improvement from the 4 to 12-months follow-up. Logistic regression model showed that female gender (*p* = 0.006), arterial hypertension (*p* = 0.01), obesity (0.04), anxiety (*p* < 0.0001), and depressive (*p* = 0.02) symptoms at 4-months follow-up were associated with persistence of anxiety symptoms at 12 months. At logistic regression analysis female gender (*p* = 0.02) and depressive symptoms at 4-months follow-up (*p* = 0.01) were associated with depressive symptoms after 12 months.

**Conclusion:**

Severity of the disease in the acute phase, in this study, was not a determining factor in identifying subjects at risk of developing clinically relevant anxiety and depression as a consequence of COVID-19 disease. Findings from the logistic regressions suggest that the factors most affecting depression and anxiety in COVID survivors after 12 months were female gender, the presence of anxiety and depression after 4 months and some physical symptoms, not necessarily COVID-related. Impact of infection and consequent hospitalization for COVID-19 did no longer represent a relevant issue for depressive symptoms, compared to other general factors.

## Introduction

It is now widely acknowledged that several systems, including the central nervous (CNS) one, may be interested by COVID-19 infection and sequelae, beyond the most common respiratory clinical features (1–7). The term “Long COVID” has been coined to describe people recovered from COVID-19, but still having lasting symptoms 28 days or more after the clinical onset of the infection (e.g., fatigue, muscle weakness, sleep difficulties, anxiety or depression) (8, 9). Beside physical issues, mental health-related symptoms can persist over time, as already observed in previous coronaviruses outbreaks (10, 11).

A recent meta-analysis estimated the pooled prevalence of mental disorders among COVID-19 survivors (12). A high heterogeneity of the 27 studies included was highlighted, but it clearly emerged that psychological and mental health sequalae were a relevant issue for COVID-19 survivors. The following prevalence rates were reported: 20% (95% CI = 16–24%) for post-traumatic stress disorder (PTSD), 22% (95% CI = 18–27) for anxiety, 36% (95% CI = 22–51%) for psychological distress, 21% (95% CI = 16–28%) for depression, and 35% (95% CI = 29–41%) for sleeping disorders. An increased risk of anxiety, depression, or overall mental health sequelae was linked to the following risk factors: inflammatory markers (especially IL-6) (13), disease severity, symptoms duration, illness severity at 6 months follow-up, female sex (14–16).

A recent cohort study involving individuals registered at an English primary care practice during the pandemic, suggested the possible role of confounding factors for the positive association between COVID-19 and psychiatric morbidity (17). Furthermore, there are suggestions about the possible role of COVID-19 treatments in the onset and/or exacerbation of mental disease, beyond the direct/indirect one of SARS-CoV-2 infection (e.g., virus neurotropism, immune response to SARS-CoV-2, hypothalamic-pituitary-adrenal axis hyperactivity, increased stress levels, and neuroinflammation) (14, 18).

Although mid- and long-term psychiatric outcomes of individuals recovered from COVID-19 are predicted, (8, 19–23) the available literature is highly heterogeneous (sample size, inclusion and exclusion criteria, follow-up duration); furthermore, patients’ assessment is based mainly on different assessment tools and self-administered questionnaires and not on a clinical diagnosis (19, 22–24). For this reason, a deeper understanding of the heterogeneity of long COVID is still warranted.

In the context of a multi-disciplinary follow-up project, we already investigated the mid-term psychiatric outcomes of the infection in a sample of 238 patients hospitalized and then recovered from COVID-19 (25). According to our hypothesis, we found that the persistence of physical symptoms (especially respiratory ones) at 4-months follow-up, rather than the severity of the acute disease, was the main driver in the maintenance of the mid-term mental health consequences of the COVID-19 infection.

Follow-up proceeded afterward and patients were re-assessed after 1-year since hospital discharge (26). The same cohort involved in the 4-months follow-up was contacted again, and 200 out of the 238 patients who already participated accepted the 12-months follow-up clinical assessment. At least one persistent symptom was reported by 79 patients (39.5%); diffusing capacity of the lungs for carbon monoxide (DLCO) < 80 and < 60% was observed in 96 (49.0%) and 20 patients (10.2%), respectively. A certain degree of motor impairment was observed in 25.8% of subjects and 37 patients (18.5%) showed moderate-to-severe persistent PTS symptoms. Overall, in our cohort, an improvement emerged from the 4 to 12-months follow-up in motor function, but not in the respiratory one (26).

As for the 4-months follow-up, the psychiatric assessment included both a thorough clinical interview performed by an experienced psychiatrist and self-administered questionnaires. We investigated the possible association of the long-term mental health consequences of the COVID-19 infection with patients’ current clinical status, persistent physical impairment, and severity of acute phase of the disease. We expected to find a reduction of the mental health consequences and that these, as for the 4-months follow-up, would be associated with the persistence of other long-lasting symptoms.

## Materials and Methods

We designed a prospective study including patients who were admitted for COVID-19 infection to the COVID Wards of the University Hospital “AOU Maggiore della Carità” in Novara (Northern Italy), and eventually discharged from the hospital between 1st March 2020 and 29th June 2020. As previously reported (9), 238 out of 767 patients consented to participate in a 4-months (131 [IQR: 119–145 days]) follow-up visit (T1). The only exclusion criterion applied was the unwillingness to provide written informed consent (see Ref 9, 26 for further details about the enrollment procedure).

The detailed results of the 4-month follow-up assessment of mental health outcomes are described elsewhere (25). At 12 months (366 [IQR: 363–369 days]) (T2), 200 out of these 238 COVID survivors accepted to take part in the assessment again [31 patients declined participation, 1 patient was excluded because she was pregnant, and 6 patients were lost at the follow-up, see Bellan et al. (26)]. Out of the 200 patients of the eligible population psychiatric assessment data were available for 196 (97.5%).

The study protocol was approved by the local ethical committee (Comitato Etico Interaziendale Novara; IRB code CE 117/20) and research was conducted according to the principles of the Declaration of Helsinki. Clinical data collection was conducted using the Research Electronic Data Capture software (REDCap, Vanderbilt University), and a unique pseudonymized code was attributed to each patient included in the study.

For each patient, we gathered information about the following: socio-demographic characteristics (age, gender, smoking attitude), comorbidities (history of chronic obstructive pulmonary disease - COPD-, hypertension, diabetes, ischemic cardiac disease, obesity), home medications, symptoms during the acute phase, complications during the hospital stay, type of oxygen support during hospitalization, and intensive care unit (ICU) admission.

Moreover, the multi-disciplinary team offered patients a thorough assessment on an outpatient basis. Participants were assessed in the hospital setting, as follows: internal medicine visit, pneumological visit including spirometry (the forced expiratory volume in the 1st second FEV1 and the forced vital capacity FVC were recorded), physiatric visit with a physical performance test and psychiatric visit, including a psychiatric interview and self-administered questionnaires. Complaints about persistent dyspnea and the perceived tolerance of physical efforts were recorded.

As far as the psychiatric assessment is concerned, we used both clinical interviews and self-administered measures in order to have both objective and subjective evaluations, which has already proved a more complete and exhaustive approach than those relying on questionnaires alone (25).

Patients were interviewed by an experienced psychiatrist, trained in the use of the Mini-International Neuropsychiatric Interview (MINI) (27), using structured and unstructured questions about current mental health status. Information about previous psychiatric history (being already treated by psychiatric services; a history of depression and/or anxiety), the presence/absence of depressive and anxiety symptoms (independent of a full-criteria diagnosis of major depressive disorder, panic disorder, and generalized anxiety disorder), and changes in sleep and eating patterns after discharge from the hospital was gathered. The outcome of the psychiatric consultation (no further indication; referral to further psychiatric care for support or medication) was recorded. Furthermore, patients were asked to fill in the following self-administered measures: Beck Depression Inventory (BDI-II), Beck Anxiety Inventory (BAI), Resilience Scale for Adults (RSA), and Impact of Event Scale (IES). The COVID-19 Peritraumatic Distress Index (CPDI) was administered at T2 (while it was not available at T1). The validated Italian version was used for all the self-administered measures.

### The Mini-International Neuropsychiatric Interview

Brief structured interviews based on DSM-IV and ICD-10 with the aim of being short, simple, but highly sensitive tools. The administration time is approximately 15 min. For this study, as in the 4-months follow-up, we adopted the A, E, and O modules to screen for depressive, panic, and generalized anxiety symptoms (27).

### Beck Depression Inventory

A self-report questionnaire with 21 items rated on a 4-points scale (from 0 to 3), asking about depressive symptoms in the last 2 weeks. Total scores identify different levels of depression: minimum (0–13); mild (14–19); moderate (20–28); and severe (29–63). Internal consistency measured by Cronbach Alpha is 0.86 for the mental component and 0.65 for the somatic component. For the subsequent analyses, we subdivided participants into two groups: those with a total score higher than 13 (mild-moderate-severe) and those with minimum BDI-II scores (28, 29).

### Beck Anxiety Inventory

A self-report questionnaire composed of 21 items measuring the emotional, physiological, and cognitive symptoms of anxiety as well as its severity. Each item is rated on a 4-points scale (from 0 to 3). Total scores identify different levels of anxiety: minimum (0–21); moderate (22–35); high (> 36). BAI internal consistency as measured by Cronbach Alpha is 0.94. For the subsequent analyses, we categorized participants into those with minimum BAI scores and those with higher scores (moderate–severe) (30, 31).

### Resilience Scale for Adults

A self-administered scale composed of 33 items examining intra- and inter-personal protective factors, that facilitate adaptation in front of psychosocial adversity. Resilience can be divided into six subscales: positive perception of self, positive perception of the future, social competence, structured style, family cohesion, and social resources. There are no cut-offs; the higher the total score, the greater are resilience levels. Internal consistency evaluated by Cronbach’s alpha is 0.86 (32, 33).

### Impact of Event Scale

The IES is a 15 items self-reported 4-point scale based on how often an event has occurred in the past week (0 not at all; 1, rarely; 3, sometimes; 5, often), to assess the presence of post-traumatic stress (PTS) symptoms. The IES identifies a total subjective stress score and 2 subscales, one measuring intrusive symptoms (intrusive thoughts, nightmares, intrusive feelings, and imagery), with scores ranging from 0 to 35, and the other measuring avoidance symptoms (numbing of responsiveness and avoidance of feelings, situations, or ideas), with scores ranging from 0 to 40. The internal consistency coefficient for intrusion is 0.84, for avoidance it is 0.71 (34, 35).

### COVID-19 Peritraumatic Distress Index

A self-administered, 24-item questionnaire designed in China to assess peritraumatic distress symptoms in relation to COVID-19 pandemic. Patients rate each item on a 5-point scale, ranging from 0 (never) to 4 (most of the time). The CDPI investigates the presence of anxiety, depression, specific phobias, cognitive change, avoidance and compulsive behavior, physical symptoms, and loss of social functioning during the past week. The total score indicates the distress level: mild to moderate distress (28–51), severe distress (≥ 52). The Cronbach’s alpha of the CPDI is 0.95 (36, 37).

### Statistical Analysis

Data were analyzed using the Stata statistical software version 15.1 (38). Normality was assessed by Shapiro–Wilk test. The measures of centrality and dispersion chosen for continuous variables were medians and interquartile ranges (IQRs). Categorical variables, whenever dichotomous or nominal, were reported as frequencies and percentages.

The results of tests and clinical interviews have been compared between different time-points by Wilcoxon’s test for paired samples and McNemar’s test (when reported as categorical). Independent variables analyzed with Wilcoxon’s test were IES, RSA, BAI, BDI; those analyzed with McNemar’s test were BAI (categorized by severity), BDI (categorized by severity), presence/absence of anxiety symptoms, presence/absence of depressive symptoms, presence/absence of changes in sleep patterns, and presence/absence of changes in eating patterns.

To test the association between the persistence of anxiety and depressive symptoms (evaluated both with the clinical interview and the questionnaires BAI and BDI-II, respectively) at 12 months with relevant continuous and categorical variables, we used the Mann–Whitney test and the Pearson χ^2^ (or Fisher exact test, as appropriate), respectively. The following continuous variables were evaluated: age, body mass index (BMI), cumulative illness rating scale (CIRS), diffusing lung capacity for carbon monoxide (DLCO), length of hospital stay, total lung capacity (TLC), forced vital capacity (FVC), forced expiratory volume in the 1st second (FEV1), IES, RSA, CPDI. We also evaluated the following categorical variables: history of anxiety and/or depression, ongoing treatment by psychiatric services, smoking attitude, sleep and appetite problems at 12-months follow-up, anxiety and/or depression at the 4-months follow-up evaluation, gender, comorbidities (diabetes, arterial hypertension, chronic obstructive pulmonary disease, ischemic heart disease, and obesity) persistence of dyspnea or exercise intolerance/poor tolerance to physical efforts at 12-months follow-up, compromised DLCO, severity of the acute phase of disease/need for intensive care unit admission.

Finally, we used logistic regression analysis to identify potential predictors of symptomatic anxiety and/or depression at 1 year; to do so, we included all variables that at univariate analysis had a *p*-value ≤ 0.2 for an association with persistent symptoms. As we were interested in identifying potential predictors, we included in the analysis only those variables which could act as such, and hence excluded those measured at 12 months.

To deal with a complete separation issue, we built the corresponding logistic models using a penalized maximum likelihood estimation approach (Firth’s logit) (39).

*P*-values were 2-sided, and statistical significance was set at *p* = 0.05.

## Results

At the 12-months follow-up, we recruited 200 patients, for 196 (males 120, 61.2%) of whom data from the psychiatric assessment were available; patients’ median age was 61.5 years [IQR: 51.0–70.5] and their median BMI was 27.5 [IQR: 24.6–31.6] kg/m^2^. The overall median length of in-hospital stay was 9.0 days [IQR: 5.0–16.0]. During the acute phase of the disease, 23 (11.7%) patients were admitted to the ICU for a median of 10.0 [IQR: 6.0–21.5] days.

The main features of the 12-months follow-up sample and their main comorbidities have been described in detail elsewhere (26).

After 12 months since discharge, patients still complained of the following symptoms: reduced tolerance to physical exercise (48%), alopecia (36.2%), fatigue (33.7%), dyspnea (34.2%), arthromyalgia (21.9%), asthenia (15.4%), cough (11.2%), anosmia (9.7%), and dysgeusia (6.6%). The diffusion capacity of the lung for carbon monoxide (DLCO) at 12-months was as follows: ≥ 80% in 50.3%, 60–79% in 39.4%, < 60% in 10.4% of patients.

Eight of the patients assessed (4.1%) reported a clinical history of anxiety or depression, 30 (15.4%) had a previous contact with psychiatric services in their medical history, and 25 (12.8%) patients were currently being treated by psychiatric services.

### Self-Administered Questionnaires

The CPDI was used only at the 12-month follow-up, and its median score was 9 [IQR: 4–18], with higher values in females (8 [3–16] vs. 12 [5–26]; *p* = 0.02).

Results of other self-administered questionnaires were compared with those obtained at the 4-month follow-up visit, with no statistically significant difference in their median values. The IES median score at 4 months was 6 [IQR: 1–19] and at 12 months was 7 [IQR: 1–20] (*p* = 0.79); the RSA median score at 4 months was 137 [IQR: 117–151] and at 12 months was 133 [IQR: 117–146] (*p* = 0.26). The BAI median score was 4 [IQR: 1–9] and 3 [IQR: 0–9] (*p* = 0.74) at the 4-months and at the 12-months follow-up, respectively; the BDI median score was 3 [IQR: 0–8] and 2 [IQR: 0–7] (*p* = 0.63) at the 4-months and at the 12-months follow-up, respectively.

Furthermore, the comparison between the two follow-up moments did not yield any statistically significant difference between either the BAI or BDI scores categorized according to severity. With more detail, BAI high-scorers (score > 21) were 14 (7.2%) and 11 (5.6%) (*p* = 0.69), while BDI high scorers (score > 13) were 24 (12.3%) and 27 (13.8%) (*p* = 0.69), at the 4- and 12-months follow-up, respectively.

When comparing males and females, the latter scored significantly higher on the IES (5 [1–16] vs. 8 [3–26]; *p* = 0.02), BAI (2 [0–7] vs. 4.5 [1.5–14]; *p* = 0.02), and BDI (1.5 [0–5] vs. 4 [0–12]; *p* = 0.02), while there was no difference in resilience (the RSA score did not differ between groups, *p* = 0.20).

### Clinical Interview

At the clinical interview, performed with the aid of the MINI, anxiety and depressive symptoms were still present in 33 (16.9%) and 37 (19.1%) of participants at the 12-months follow-up, whereas they were found in 67 (34.5%) and 62 (32.1%) patients at the 4-months follow-up, respectively.

A change in the sleep pattern emerged in 61 (31.3%) patients at 4-months and persisted in a lower number of subjects at the 12-months follow-up (*N* = 31, 15.9%; *p* < 0.01). At 12 months, a change in the sleep pattern was more common in females (*N* = 18, 23.7%) than in males (*N* = 13, 10.9%; *p* = 0.03). Finally, a reduction in appetite was reported by 33 patients (16.9%) at 4-months but improved at the 12-month visit (*N* = 12, 6.2%; *p* < 0.01), with no differences between genders (*p* = 0.22).

#### Anxiety Symptoms

Anxiety levels registered at the clinical interview showed a significant improvement from the 4 to 12-months follow-up (*p* < 0.01).

The results of the univariate analysis to test the association between the persistence of anxiety symptoms and potentially relevant clinical and demographic variables are reported in Tables 1, 2. The logistic regression model (Table 3) showed that female gender (*p* < 0.01), arterial hypertension (*p* = 0.01), obesity (*p* = 0.04), anxiety (*p* < 0.01), and depressive (*p* = 0.02) symptoms at the 4-months follow-up visit were independently associated with the persistence of anxiety symptoms at 12 months.

**TABLE 1:** Comparison of independent samples.

	No anxiety at the 12-months follow-up *N* = 163	Anxiety at the 12-months follow-up *N* = 33	*P*
Age, years	62 [51–70]	58 [49–71]	0.57
BMI, Kg/m^2^	27.5 [25.6–31.3]	29.4 [24.8–34]	0.33
CIRS	2 [1–3]	2 [2–2]	0.49
DLCO_12M	80 [69–91]	76 [67–87]	0.47
Length of stay	9 [5–16]	9 [5–18]	0.75
FEV1_12M	102 [91–113]	96 [91–108]	0.27
FVC_12M	98 [90–110]	97 [89–107]	0.62
TLC_12M	102 [94–107]	101 [94–104]	0.66
IES_Tot_12M	5 [1–16]	18 [8–37]	**<0.0001**
CPDI	8 [4–15]	18 [8–38]	**0.0010**
RSA_TOT_12	141 [119–153]	126 [108–141]	**0.0030**

*Continuous variables associated with anxiety as assessed with the clinical interview at the 12-month follow-up.*

*Statistically significant results are in bold. In square brackets IQR InterQuartile Range.*

*BMI, Body Mass Index; CIRS, Cumulative Illness Rating Scale; CPDI, COVID-19 Peritraumatic Distress Index; DLCO, Diffusion capacity of the Lung for Carbon Monoxide; FEV1, forced expiratory volume in the 1st second; FVC, Forced Vital Capacity; IES, Impact of Event Scale; RSA, Resilience Scale for Adult; TLC, Total Lung Capacity.*

**TABLE 2:** Anxiety and depression as assessed with the psychiatric interview at the 12-month follow-up: association with psychiatric, somatic, and clinical variables (chi-squared test).

		Anxiety at the clinical interview 12-months follow-up		*p*	Depression at the clinical interview 12-months follow-up		*p*
		Yes N (%)	No N (%)		Yes N (%)	No N (%)	
History of anxiety and/or depression	*Yes*	3 (37.5)	5 (62.5)	0.14	4 (50.0)	4 (50.0)	**0.04**
	*No*	30 (16.1)	157 (83.9)		33 (17.7)	153 (82.3)	
Already treated by psychiatric services	*Yes*	11 (36.6)	19 (63.4)	**0.006**	14 (46.6)	16 (53.4)	**<0.001**
	*No*	22 (13.4)	142 (86.6)		23 (14.1)	140 (85.9)	
Sleep problems at 12-months follow-up	*Yes*	15 (48.3)	16 (51.7)	**<0.001**	19 (61.3)	12 (38.7)	**<0.0001**
	*No*	18 (10.9)	146 (89.1)		18 (11.0)	145 (89.0)	
Appetite problems at 12-months follow-up	*Yes*	6 (50.0)	6 (50.0)	**0.006**	8 (66.7)	4 (33.3)	**0.0002**
	*No*	27 (14.8)	156 (85.2)		29 (15.9)	153 (84.1)	
Anxiety at the clinical interview, 4-months follow-up	*Yes*	27 (40.2)	40 (59.8)	**<0.0001**	26 (38.8)	41 (61.2)	**<0.0001**
	*No*	6 (4.7)	121 (95.3)		11 (8.7)	115 (91.3)	
Depression at the clinical interview, 4-months follow-up	*Yes*	24 (38.1)	39 (61.9)	**<0.0001**	26 (41.9)	36 (58.1)	**<0.001**
	*No*	9 (6.9)	122 (93.1)		11 (8.4)	120 (91.6)	
Gender	*Male*	10 (8.4)	109 (91.6)	**<0.001**	12 (10.2)	106 (89.8)	**0.0001**
	*Female*	23 (30.3)	53 (69.7)		25 (32.9)	51 (67.1)	
Diabetes	*Yes*	4 (13.3)	26 (86.7)	0.79	4 (13.3)	26 (86.7)	0.46
	*No*	29 (17.6)	136 (82.4)		33 (20.1)	131 (79.9)	
Hypertension	*Yes*	19 (23.4)	62 (76.6)	**0.05**	17 (20.9)	64 (79.1)	0.58
	*No*	14 (12.2)	100 (87.8)		20 (17.6)	93 (82.4)	
COPD	*Yes*	3 (25.0)	9 (75.0)	0.43	4 (33.3)	8 (66.7)	0.25
	*No*	30 (16.3)	153 (83.7)		33 (18.1)	149 (81.9)	
Ischemic heart disease	*Yes*	0 (0)	18 (100.0)	**0.05**	0 (0)	18 (100)	**0.03**
	*No*	33 (18.6)	144 (81.4)		37 (21.0)	139 (79.0)	
Exercise intolerance/poor tolerance to physical efforts at 12-months follow-up	*Yes*	24 (25.5)	70 (74.5)	**0.002**	29 (30.9)	65 (69.1)	**<0.0001**
	*No*	9 (8.9)	92 (91.1)		8 (8.0)	92 (92.0)	
Dyspnea at 12 months follow-up	*Yes*	18 (26.8)	49 (73.2)	**0.009**	20 (29.9)	47 (70.1)	**0.007**
	*No*	15 (11.7)	113 (88.3)		17 (13.4)	110 (86.6)	
DLCO < 60%	*Yes*	5 (25.0)	15 (75.0)	0.35	5 (25.0)	15 (75.0)	0.54
	*No*	28 (16.3)	144 (83.7)		31 (18.1)	140 (81.9)	
DLCO < 80%	*Yes*	18 (18.8)	78 (81.2)	0.70	17 (17.8)	78 (82.2)	0.85
	*No*	15 (15.6)	81 (84.4)		19 (19.7)	77 (80.3)	
ICU admission	*Yes*	3 (13.0)	20 (87.0)	0.77	3 (13.0)	20 (87.0)	0.58
	*No*	30 (17.4)	142 (82.6)		34 (19.8)	137 (80.2)	
Clinical severity	*Class 3*	10 (22.2)	35 (77.8)	0.74	9 (20.0)	36 (80.0)	0.45
	*Class 4*	1 (11.1)	8 (88.9)		1 (11.1)	8 (88.9)	
	*Class 5*	13 (17.1)	63 (82.9)		13 (17.3)	62 (82.7)	
	*Class 6*	7 (15.9)	37 (84.1)		12 (27.3)	32 (72.7)	
	*Class 7*	2 (9.5)	19 (90.5)		2 (9.5)	19 (90.5)	
Obesity	*Yes*	8 (38.1)	13 (61.9)	**0.01**	7 (33.3)	14 (66.7)	0.14
	*No*	25 (14.4)	149 (85.6)		30 (17.3)	143 (82.7)	
Smoking	*Never*	21 (19.4)	87 (80.6)	0.58	20 (18.7)	87 (81.3)	0.97
	*Current*	3 (13.6)	19 (86.7)		4 (18.2)	18 (81.8)	
	*Former*	9 (13.8)	56 (86.2)		13 (20.0)	52 (80.0)	

*Statistically significant results are in bold.*

*Class 3: Hospitalized, not requiring supplemental oxygen and no longer requiring ongoing medical care.*

*Class 4: Hospitalized not requiring supplemental oxygen but requiring ongoing medical care.*

*Class 5: Hospitalized requiring any supplemental oxygen.*

*Class 6: Hospitalized requiring non-invasive ventilation or use of high-flow oxygen devices.*

*Class 7: hospitalized, receiving invasive mechanical ventilation or extracorporeal membrane oxygenation (ECMO).*

*COPD, chronic obstructive pulmonary disease; DLCO, Diffusion capacity of the Lung for Carbon Monoxide; ICU, Intensive Care Unit; TLC, Total Lung Capacity.*

**TABLE 3:** Logistic regression model—anxiety at 12 months.

Variable	Odds ratio (95%CI)	*P*-value
Female gender	4.05 (1.51–11.02)	**0,006**
Arterial Hypertension	3.63 (1.32–10.07)	**0.01**
Obesity	3.49 (1.06–11.47)	**0.04**
Ischemic heart disease	0.31 (0.01–6.75)	0.45
History of anxiety and/or depression	0.76 (0.14–4.06)	0.75
Already treated by psychiatric services	0.64 (0.21–1.99)	0.45
Anxiety at the clinical interview, 4-months follow-up	9.11 (2.77–29.96)	**<0.0001**
Depression at the clinical interview, 4-months follow-up	3.59 (1.21–26.31)	**0.02**

*Statistically significant results are in bold.*

#### Depressive Symptoms

Depressive symptoms at the clinical interview improved significantly from the 4 to 12-months follow-up (*p* < 0.01).

The results of the univariate analysis testing the association between the persistence of depressive symptoms and potentially relevant clinical and demographic variables are reported in Tables 2, 4. Female gender (*p* = 0.02) and depressive symptoms at the 4-months follow-up visit (*p* = 0.01) were associated with the persistence of depressive symptoms at 12 months at the logistic regression analysis (Table 5).

**TABLE 4:** Comparison of independent variables.

	No depression at the 12-months follow-up *N* = 159	Depression at the 12-months follow-up *N* = 37	*P*
Age, years	62 [51–70]	61 [49–71]	0.99
BMI, Kg/m^2^	27.2 [24.6–31.2]	30.4 [24.9–33.4]	0.08
CIRS	2 [1–3]	2 [2–2]	0.87
DLCO_12M	79 [70–91]	81 [66–88]	0.91
Length of stay	9 [5–16]	10 [5–15]	0.91
FEV1_12M	102 [92–113]	98 [88–109]	0.24
FVC_12M	98 [91–109]	96 [84–113]	0.5
TLC_12M	102 [94–107]	101 [94–108]	0.56
IES_Tot_12M	5 [1–16]	23 [6–40]	**0.0001**
CPDI	8 [3–15]	18 [9–37]	**0.0001**
RSA_TOT_12	141 [122–153]	120 [99–108]	**0.0001**

*Continuous variables associated with depression as assessed with the clinical interview at the 12-month follow-up.*

*Statistically significant results are in bold. In square brackets IQR InterQuartile Range.*

*BMI, Body Mass Index; CIRS, Cumulative Illness Rating Scale; CPDI, COVID-19 Peritraumatic Distress Index; DLCO, Diffusion capacity of the Lung for Carbon Monoxide; FEV1, forced expiratory volume in the 1st second; FVC, Forced Vital Capacity; IES, Impact of Event Scale; RSA, Resilience Scale for Adult; TLC, Total Lung Capacity.*

**TABLE 5:** Logistic regression model: depression at 12 months.

Variable	Odds ratio (95%CI)	*P*-value
Female gender	2.69 (1.15–6.34)	**0.02**
Arterial hypertension	1.54 (0.65–3.67)	0.33
Obesity	2.28 (0.75–6.90)	0.15
Ischemic heart disease	0.20 (0.01–3.81)	0.28
History of anxiety and/or depression	1.39 (0.28–6.96)	0.69
Already treated by psychiatric services	1.41 (0.50–3.96)	0.51
Anxiety at the clinical interview, 4-months follow-up	2.39 (0.88–6.50)	0.09
Depression at the clinical interview, 4-months follow-up	3.46 (1.34–8.94)	**0.01**

*Statistically significant results are in bold.*

## Discussion

Mid- and long-term psychiatric outcomes of COVID-19 infection are predicted (8, 19–23), and the available literature suggests that psychological and mental health sequalae are a relevant issue for recovered patients (12). As for the first step of our follow-up research, we assessed patients both with a thorough clinical interview performed by an experienced psychiatrist and with self-administered questionnaires.

The presence of anxiety and depression at the psychiatric interview carried out 12-months after discharge affected 16.9 and 19.1% of the patients assessed, respectively. Our percentages are slightly lower than those reported by a recent meta-analysis (22 and 21% for anxiety and depression, respectively), nonetheless the timing and procedure of the assessment could account for these differences (12). As expected, according to the study hypothesis, the 12-months percentages we found were in reduction compared to the first follow-up step, carried out 4 months after discharge, in which anxiety and depression were found in 34.5 and 32% of the subjects, respectively. This trend seems to suggest that the impact of COVID-19 hospitalization on anxious and depressive symptoms globally decreases over time. Nonetheless, it must be considered that in such a long period, many additional and confounding factors, impossible to assess, might have occurred. We cannot exclude that this improvement could be attributed, at least in part, to participation in the study itself; it is acknowledged that participating in similar studies could offer patients the perception of greater care, thus mitigating the impact of COVID-19 on mental health sequelae, being part of the placebo effect (40). Furthermore, selection and participation bias should be accounted for, as it is reasonable to assume that the subjects who decided to take part in the study were more attentive to health or suffering from less severe global sequelae.

On the other hand, as can be seen from the analysis of the self-administered questionnaires (IES for stress, BAI for anxiety, and BDI for depression), none were found to change significantly from 4 to 12 months. The discrepancy between self-assessment and clinical interview already emerged at the 4-months follow-up, thus supporting our previous suggestion that a twofold approach could be more complete and more sensible in identifying psychiatric symptoms, as no self-administered questionnaire can equate to a clinical diagnosis (25).

It is noticeable that the rate of depressive symptoms percentages we identified in our patients is comparable to those reported by the general population in a recent international multicenter study (41), which evaluated the presence of depression during the pandemic with online questionnaires. Therefore, this would seem to suggest that in our sample the impact of the infection and consequent hospitalization for COVID-19 after 12 months would no longer be crucial, at least as regards depressive symptoms, compared to other general factors.

Some recent studies found overall mood and anxiety disorders rates consistent with our results, even though the timing and modality of the assessments performed should be accounted for Taquet et al. (42). Our results failed to support the finding of a higher risk of anxiety or depression in patients with more severe illness at 6 months follow-up (14, 43, 44). Nonetheless, it is necessary to consider different sample sizes and that, in our study, we assessed only patients who agreed to participate in the multidisciplinary evaluation.

We identified a significant association between the presence of anxiety and depression at the 12-months follow-up clinical interview and the persistence of dyspnea and impaired exercise tolerance, in line with our previous findings (25) and with literature data supporting an association between depression and/or anxiety, the persistence of respiratory distress (45), and other physical symptoms (46) in the context of the long COVID clinical picture.

Moreover, the presence of anxiety and depression at the 4 and 12-month follow-up psychiatric interview was significantly correlated with having a previous history of anxiety and depression and being already treated by psychiatric services (25).

Moreover, higher levels of IES total score, of CPDI total score and lower levels of RSA total score were found in the group of patients with anxiety and depression as assessed at the 12-month follow-up psychiatric interview, compared to the groups with no anxiety and no depression, respectively. Greater resilience would therefore seem associated with a better outcome with regard to the mental health sequelae related to a previous hospitalization for COVID-19. This finding is supported by a study involving a sample of the general population during the pandemic, which proposed a model where resilience mediated the negative effects of stress on anxious and depressive symptoms (47). On the contrary, CPDI and IES total scores were directly correlated to levels of anxiety and depression, thus suggesting and intertwining between the persistence of depressive and anxious symptoms and the traumatic impact that the event had on patients.

The logistic regression model showed that patients of female sex, with clinically assessed anxiety and depression after 4 months, with arterial hypertension and obesity, showed greater odds of having anxiety at the 12-month follow-up psychiatric interview. Considering depression, in the logistic regression model, patients of female sex and with clinically assessed depression after 4 months showed greater odds of having depressive symptoms at the 12-month follow-up psychiatric interview.

Contrary to what we would expect according to our *a priori* hypothesis, the presence and persistence of one or more COVID-related organic comorbidities was not associated with the finding of anxious-depressive symptoms at the psychiatric interview (except for arterial hypertension and obesity). This data is in contrast with the findings of our 4-months follow-up study and with what seems to emerge from the available literature, notwithstanding its high heterogeneity. Indeed, not only is the presence of chronic diseases associated with anxiety and depression in the general population (48) but also this would also seem true for COVID-19 patients (49). Our results seem to suggest that in patients, after 12 months since discharge from the hospital, the factors that most affect anxiety symptoms may be some physical symptoms (such as obesity and hypertension), not necessarily related to their previous COVID-19 infection. Indeed, scientific literature highlights that both obesity and arterial hypertension are associated with increased levels of anxiety (50, 51). It could be hypothesized that in the population we examined, the greater impact of the traumatic event (the COVID-19 infection) may have reduced the effect of other conditions usually associated with anxious-depressive symptoms. Another hypothesis is that the selection bias eventually yielded a sample of subjects with a greater propensity to request health support.

In this study, female sex had greater odds of having anxiety at the psychiatric interview. This increased susceptibility is corroborated by the literature (52). Female sex has been identified as a risk factor for the development of psychiatric symptoms in the specific population of subjects with previous COVID-19 infection (49).

According to the CPDI, which we administered only at the 12-month follow-up, a median score of 9 was found, proving no distress in our sample. It must be taken into consideration the specific period when this test was administered. Different results, with considerably higher levels of peritraumatic distress, were found in our country during lockdown in a study involving the general population (53).

In our sample, the persistence of changes in appetite and sleep patterns at 12 months was significantly correlated with the presence of anxiety and depression. These symptoms are themselves characteristics of depression and anxiety, therefore there is likely an overlap, at least partial, of these features.

In general, studies on this topic are highly heterogeneous (in terms of sample selection, diagnostic tools, and follow-up approach), making it difficult to compare results from different studies (45).

## Limitations

The first limitation of this study is that it is monocentric, which reduces both the number and variety of subjects involved. The second is the possible participation and selection bias, as 80% of the patients who had participated in the study at 4 months also participated at 12 months, while the starting sample was of 732 patients. On the one hand, only patients in whom the impact of the trauma was minor and for whom therefore presenting to the hospital after admission did not represent an unbearable re-enactment of the traumatic event could have participated. On the other hand, subjects with a greater psycho-physical impairment, who would have seen the outpatient appointment as a possibility of support, could have mainly participated. Unfortunately, as with other similar studies, we did not have pre-COVID-19 baseline data relating to the mental health of the examined sample. In this article, we did not consider cytokines and inflammatory markers and their correlation with depression. Unfortunately, albeit we included a measure of resilience, we did not focus on post-traumatic growth (PTG) or potentially positive psychological changes in response to challenging life circumstances, as it may have had an impact on the 1-year improvement of mental health symptoms, as suggested by literature (54, 55). However, during the clinical evaluation, we collected data relating to the psychiatric history and previous contacts with mental health services. If the use of self-administered tests might represent a limitation of studies similar to ours, one important strength of this study was the association with a psychiatric diagnostic interview, which yielded the possibility to focus on a clinical diagnosis rather than on self-administered questionnaire scores.

Another relevant strength was that this was a multidisciplinary study: in addition to psychiatrists, internists, pulmonologists, and physiatrists evaluated patients, with a multifaceted approach.

## Conclusion

The persistence of symptoms is a primary determinant of mental health outcome, with anxiety, depression, sleep disturbances, and post-traumatic stress symptoms being commonly reported in patients recovered from COVID-19.

As in our previous 4-months follow-up study, the severity of the disease in the acute phase did not seem to be a determining factor in identifying subjects particularly at risk of developing clinically relevant anxiety and depression because of COVID-19 disease. Among patients hospitalized for COVID-19, subjects with a previous psychiatric diagnosis and those of female sex were particularly vulnerable to negative consequences on mental health. These data, confirmed by literature, suggests the importance of maintaining particular attention to the prevention and treatment of psychiatric symptoms in these subpopulations.

Given the high number of COVID-19 infections and the frequency with which multiple sequelae occur, it is important to continue studies focused on this topic, both increasing the sample number and lengthening the follow-up period even beyond the year.

## Data Availability Statement

The raw data supporting the conclusions of this article will be made available by the authors, without undue reservation.

## Ethics Statement

The studies involving human participants were reviewed and approved by Comitato Etico Interaziendale Novara; IRB code CE 117/20. The patients/participants provided their written informed consent to participate in this study.

## NO-MORE COVID Group

Gian Carlo Avanzi, Giulia Baldon, Marco Battaglia, Sofia Battistini, Emanuela Cadario, Vincenzo Cantaluppi, Giuseppe Cappellano, Luigi Mario Castello, Annalisa Chiocchetti, Federico Ceruti, Elisa Clivati, Simona De Vecchi, Martina Gai, Francesco Gavelli, Mara Giordano, Leonardo Grisafi, Marco Invernizzi, Marcello Manfredi, Paolo Marzullo, Francesco Murano, Elena Parachini, Filippo Patrucco, Giuseppe Patti, Roberto Piffero, Davide Raineri, Cristina Rigamonti Roberta Rolla, and Iris Zeqaj.

## Author Contributions

MP, MB, AB, PB, PS, and PZ designed the study. CG, EGt, EGm, VB, AF, AJ, DM, PP, and the NO MORE COVID GROUP recruited and assessed patients. MB performed the statistical analyses. CG and PZ drafted the manuscript. All authors revised the manuscript and contributed with relevant intellectual content.

## Conflict of Interest

The authors declare that the research was conducted in the absence of any commercial or financial relationships that could be construed as a potential conflict of interest.

## Publisher’s Note

All claims expressed in this article are solely those of the authors and do not necessarily represent those of their affiliated organizations, or those of the publisher, the editors and the reviewers. Any product that may be evaluated in this article, or claim that may be made by its manufacturer, is not guaranteed or endorsed by the publisher.
